# How Do Family Role Overload and Work Interferance with Family Affect the Life Satisfaction and Sleep Sufficiency of Construction Professionals?

**DOI:** 10.3390/ijerph16173094

**Published:** 2019-08-26

**Authors:** Ruodan Lu, Ziyi Wang, Xiaoming Lin, Liang Guo

**Affiliations:** 1 School of Architecture, Building and Civil Engineering, Loughborough University, Loughborough LE11 3TU, UK; 2 Darwin College, University of Cambridge, Cambridge CB2 1TN, UK; 3 Data Science Institute and Department of Statistics, Shandong University at Weihai, Weihai 264209, China

**Keywords:** work-family conflict, sleep, family role overload, work interference with family, life satisfaction, family supportive supervision behaviors, construction professional

## Abstract

Sleep and work-family problems attract a great attention in the construction industry because construction professionals are usually prone to work-family conflicts and sleep problems. The objective of this study was to analyze the effect of Family-Role Overload (FRO) and Work Interference with Family (WIF) on sleep sufficiency. We also included life satisfaction as a mediator and family supportive supervision behaviors (FSSB) as a moderator. Using a sample of 193 Chinese construction professionals, we collected objectively-measured sleep sufficiency data with validated wrist actigraphies and self-reported sleep sufficiency data, FRO, WIF, life satisfaction and FSSB with questionnaires through multiple waves. Results demonstrated that FRO was negatively associated with both objectively-measured and self-reported sleep sufficiency and life satisfaction played an important mediating role in this relationship. The moderating effect of life satisfaction on the path between WIF and sleep sufficiency is trivial. In addition, no significant moderating effects of FSSB were found. More substantial policies should be taken to improve the life satisfaction and sleep sufficiency of construction professionals.

## 1. Introduction

Sleep problems have become an emerging global epidemic as the WHO estimates that more than one third of people around the world are sleep deprived [1]. In China, 38.2 percent of adults suffer from insomnia and more than 300 million Chinese have sleep disorders [2]. As an elementary physiological need, sleep is important to several somatic functions, including learning and memory [3], metabolism [4], and immunity [5]. Sleep deprivation can cause all kinds of internal health problems such as depression [6], obesity [7], diabetes [8] and cardiovascular disease [9]. Beyond the individual level, there is also the social side-effect of bad sleeping habits. Because sleep deprivation leads to a loss of self-control, the efficiency of teamwork will be reduced, and productivity will be cut down [10]. The necessity of adequate sleep makes it a priority to explore the antecedents of sleep insufficiency. A possible reason for sleep disorder is the emergence of family role overload and of inter-role conflict that occurs when the demands of work roles and those of family roles are mutually incompatible in some respects [11,12,13,14].

A preponderance of evidence shows that daytime stressors resulting from increasing role demands and role conflicts can significantly and negatively impact the duration and quality of sleep at night [15,16]. Once sleep debts accumulate, people feel exhausted and cannot focus at work or at home [17], which will eventually aggravate family-work interface conflicts. As an increasing number of employees and their families are suffering from this vicious circle [18], there is an urgent need of in-depth knowledge about the underlying mechanisms through which family role demands and conflicts in the work-family interface influence sleep and what are the potential mediators and moderators in the relationship.

In this article, we drew from Hobfoll’s [19] conservation of resources theory of stress as an overarching theoretical framework to suggest that family-role overload (FRO) and work interference with family (WIF) have negative impacts on sleep sufficiency through the mediation of life satisfaction. FRO is described as a family-relevant stressor, which occurs when individuals are faced with a set of obligations, tasks, responsibilities or other role functions in the family domain (i.e., housework and caregiving), which require them to do more beyond their available resources [20]. Along with excessive family role tasks, people will experience increased role strain and diminished well-being. Research reveals that heavy disability-related family care responsibilities and gender inequality in sharing household labor and caregiving cause a higher level of anxiety and increase the likelihood of depression [21,22]. Sociologists studying sleep highlight the relation between caring family members and the impacts on caregivers’ sleep primarily in a gender asymmetrical way [23], which offers the theoretical support for the negative relationship between role overload, stress and sleep. In this case, FRO is a potentially useful concept to promote a better understanding of the relationship between multiple roles and sleep.

In addition, the expectation of “ideal worker” and the popularity of workaholic behavior address more attention to the substructure called WIF. WIF occurs when experiences and commitments at work such as inflexible work hours, work overload, or an unsupportive supervisor or organization intrude on family life [24]. In today’s high-demand labor market, employers expect workers unencumbered with non-work roles to focus entirely on work. However, continuous intense pressure and heavy workloads fueled by negative management practices will generate fear and anxiety, especially when employees lack the flexibility or resources they need and feel their work is getting in the way of their families [25].

Further, some studies take the unmet personal requirements or needs as the main cause of low life satisfaction [26,27,28]. While numerous studies associate inter-role conflicts with poor life satisfaction, there are few studies evaluating the associations between life satisfaction and sleep. Life satisfaction is a long-lived cognitive evaluation of the overall quality of one’s life [29]. As life satisfaction appears to encompass many individual life domains such as social relationships, work performance and cognitive competence [28,30], it can be a determinant factor in public health research. To address the calls of Strine et al. [31], this study intended to advance the existing related studies by including life satisfaction as a mediator. Life satisfaction, the evaluation of one’s life as a whole, can be an important, yet overlooked mechanisms through which FRO and WIF influences sleep, because both are sources of stress and may influence an individual’s evaluation of quality of life, and then do harm to sleep. Apart from highlighting the key role of life satisfaction, it is also important to explore whether the dark sides of FRO and WIF can be mitigated by family supportive supervisor behaviors (FSSB). As Olson et al. [32] argued, an intervention focused on changing the workplace culture could increase the amounts of sleep employees attain both objectively and subjectively. The full FSSB training program initiated by Hammer and Kossek [33] also describes the benefits of providing emotional and instrumental support to help employees in competing work-family demands, which improves physical and mental health and leads to positive work-family spillover and job satisfaction. The stressful situation at home or at workplace could be lessened because supervisors provide more necessary resources to deal with conflicts [11].

Drawing on a sample of 193 construction professionals in Southwest China, we collected data by questionnaires and actigraphic devices. We focused on the group of construction professionals, because these people usually endure exposure to noise, dust, and unhealthy solvents and work in extremes of heat or cold, they require physical and mental energy recovered from sleep to cope with long and inflexible work hours and unsafe working conditions [34,35,36,37]. In addition, construction professionals are usually forced to endure separations from family due to the frequent and long business travel, which will trigger FRO and WIF more often and suffer higher rate of mental disorder and physical injuries than other occupational groups [38].

## 2. Theoretical Background and Hypotheses Development

Conservation of resources (COR) theory postulates that individuals are motivated to maintain their current resources and to pursue new resources [19]. Resources refer to anything that a person values and can be broken down into four categories: objects (e.g., house, phone), conditions (e.g., status, social support), personal characteristics, (e.g., optimism, self-esteem), and energies (e.g., knowledge, time). Psychological stress occurs when there is a threat of resource loss or an actual loss of resources [19,39]. According to the COR theory, FRO and WIF are particularly linked to the loss of resources such as time and positive emotions and sequentially result in psychological strain, which prevents employees from obtaining adequate sleep.

### 2.1. Family-Role Overload

Drawing from Kahn et al.’s argument [40], role overload can potentially arise in the single domain of family when an individual feels there is insufficient time or human energy to carry out all of the expected role functions within the family. In this case, individuals with higher FRO are often accompanied by stress from a lack of resources (e.g., time, ability) [41]. For example, people combining childcare with caring for older or disabled relatives are known as the sandwich generation, who are struggling to manage their time efficiently between children, older parents, family, work, and personal well-being. All of these tasks are consuming their limited time and therefore, the performance both in the family and work domains may fail to live up to their expectations. Stress can impact individuals’ well-being when individuals perceive the situation as stressful and their resources are inadequate to handle environmental stimuli (i.e., too many family-role obligations) [42]. Life satisfaction can be negatively affected by this kind of stress because life satisfaction has been associated with the attainment of a desired end and fulfillment of essential conditions [27], the process of which is in high demand of adequate resources, especially time and energy. Based on theory and research, we hypothesized the following:

**Hypothesis** **1 (H1).** *FRO is negatively associated with life satisfaction.*

### 2.2. Work Interference with Family

Three subtypes of conflict are identified within WIF: time-based, strain-based, and behavior-based [43]. Drawing from resource allocation theory [44], people need to allocate scarce resources (time) among work and family to achieve a balance goal. However, the allocation can hardly be balanced due to the conflict between heavy demands both in the work and family domains and the limited resources. First of all, time is a valuable but limited resource in one’s life. When the work domain requires more time, the time devoted to the family domain will be cut down and cannot reach the desired amount [45]. Therefore, the shortage of resource makes it difficult to perform in the family domain successfully [19]. Second, strain–based WIF entails pressures in the work role impairing performance in the family role. For instance, employees who experience strain from the workplace are likely to bring negative emotions to the family and cannot concentrate on normal family activities when they are at home [46]. And third, behavioral-based WIF states that employees who consume their precious resources to develop effective behaviors at work cannot transfer these behaviors to fulfill demands or resolve problems at home. As a result, employees feel exhausted but still annoyed by incompatible demands in their family domains. WIF eventually leads to chronic stress, burnout, and frustration, which can breed the reception of dissatisfaction with life [27]. Therefore, we hypothesized a negative relationship between WIF and life satisfaction, leading to the following:

**Hypothesis** **2 (H2).** *WIF is negatively associated with life satisfaction.*

### 2.3. Life Satisfaction and Sleep

Life satisfaction is a stable and long-lived reception, which can have a long-arm effect on physical and mental health outcomes. Strine et al. [31] investigate the direct effect of life satisfaction on sleep and argued that life satisfaction seems to independently affect sleep sufficiency regardless of other psychological stressors. Individuals who are more satisfied with life would judge that their lives fulfill their ideal life-plans. Once the life lives up to the expectation, there may be less reported worry and anxiety, which are antecedents of symptoms of sleep onset delay, wake after sleep onset [47], and even insomnia [48]. A report provided by the American Academy of Sleep Medicine [49] found that middle-aged adults who are more satisfied with their lives tend to fall asleep faster than their less satisfied peers. Therefore, they are more likely to obtain a larger amount of both self-reported and objectively-measured sleep sufficiency. On the contrast, people with low life satisfaction are more likely to put themselves on a state of stress and anxiety. While the stress raises, people cannot perform an easy sleep because the release of particularly cortisol, adrenaline and noradrenaline puts the body on a state of heightened alertness [50]. Taken together, we hypothesized the following:

**Hypothesis** **3 (H3).** *Life satisfaction is positively associated with (a) self-reported sleep sufficiency and (b) objectively-measured sleep sufficiency.*

### 2.4. Family-Supportive Supervision Behaviors (FSSB)

Hammer et al. [51] conceptualize FSSB as a multidimensional superordinate construct consisting of four subordinate dimensions: emotional support, instrumental support, role modeling behaviors, and creative work-family management. FSSB has been shown to alleviate the consequences of family role overload or inter-role conflicts [52]. Drawing on COR framework, FSSB acts as a valuable resource, providing emotional and instrumental support to help employees in competing work-family demands and providing more flexible work schedules and family-supportive assistance [53]. Employees who receive FSSB at work possess higher resources and have a greater capability to protect against resource loss, to recover from losses, and to gain new resources. The access to resources allows employees to perform better in managing multiple family role responsibilities and reduce the stress generated from work interferes with family [54]. The various work and family demands will be fulfilled better under the help of FSSB and thereby have higher level of happiness. Consequently, the negative impact of FRO and WIF on life satisfaction will be lessened.

**Hypothesis** **4 (H4).** *Family Supportive Supervision Behavior moderates (a) the relationship between FRO and life satisfaction and (b) the relationship between WIF and life satisfaction, such that these relationships are weaker for employees with a high level of Family Supportive Supervision Behaviors.*

**Figure 1:**
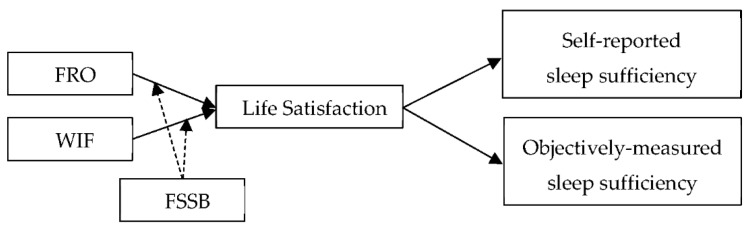
Research model.

Our research model is illustrated in Figure 1.

## 3. Materials and Measures

### 3.1. Sample

Data were collected from a large construction engineering design group in Southwest China under the help of a consulting firm. The survey was approved by the ethics committee of the corresponding institute. The group’s HR department was contacted first to explain the study purpose and permission was granted. We obtained a list of 342 engineering personnel (including architecture, structural and construction engineers, project managers and HVAC engineers) who had been working in the group for more than 12 months. We informed these engineers that the data collection would occur across multiple waves and that data collected would be used for research purposes only. They were also assured of the confidential and voluntary nature of their participation and were promised that the group would not be intervened with at all. They understood that data would be examined anonymously. Each participant who completed all waves of the questionnaire were rewarded with a 50 Yuan (around 7.42 USD) voucher. The questionnaire was translated from English to Chinese by a professional and then translated back from Chinese to English by another professional [55]. The Chinese questionnaire was then validated with 10 randomly selected graduate students before being administered to the employees. Among the 342 engineers, 193 agreed to participate in our four-wave research project. For matching reasons, every participant was assigned a distinct participant code. During the first wave of data collection, the participants signed a consent form indicating that he or she understood all the privacy risks. Then the participants completed a questionnaire asking about their perception of FRO and of WIF on site. Two weeks after the first wave, the respondents filled in the questionnaire of FSSB on site. And two weeks after the second wave, the respondents filled in the questionnaire of life satisfaction on site. They were also assigned a wristband, which should be worn every night over the next two weeks to measure objectively-measured sleep sufficiency. Finally, two weeks after the third wave, they completed the questionnaire of self-reported sleep sufficiency and returned their wristbands on site. The valid response rate was 56.43%. We compared the age and gender distributions of the participants and the non-participants and the two-sample *t*-test suggested that there were insignificant differences.

We chose a multi-wave survey design to alleviate the common method bias. That was because participants were the source for the information of FRO, WIF, life satisfaction, FSSB and self-reported sleep sufficiency, which might cause participants to bias their responses. Multi-wave survey design is a widely-used method to add temporal separation so that such procedural remedies can reduce the impact of common method variance on study results [56]. To prevent careless responses [57], we inserted an attention check question in each wave that instructed participants to circle the second to the last number shown on the survey, following the recommendation of [58]. None of the participants with fully completed data failed the attention check. Our sample was 52.3% male. Most of the participants had at least college degrees, and 78% were married. Around 73% of the participants had at least one child. The participants were aged 21–59 and their average age was 32. Although the average age of the participants was relatively young, previous studies indicated that young employees may suffer higher levels of work-family conflicts and more sleep disorders [59], therefore, our sample is appropriate for this study.

### 3.2. Measures

#### 3.2.1. Family-Role Overload (FRO)

We used a 5-item version of the scale developed by Thiagarajan at al. [60]. Consistent with [61], participants were asked to respond to a set of parallel items (i.e., in my family/personal life: I have the time and energy in general; I need more hours to fulfill the duties at home; I have time for myself; I meet expectations of family members; I have more commitments to overcome than other parents/spouses) by reflecting upon the family/home-life domain. Cronbach’s alpha was 0.92.

#### 3.2.2. Work Interference with Family (WIF)

We used a 9-item scale developed by Carlson et al. [62] to measure WIF. These items represented time (i.e., I have to spend so much time on work responsibility that I have to miss family activities, my work keeps me from my family activities and from participating equally in household responsibilities), strain (i.e., I am often too frazzled to participate in family activities/responsibilities, often emotionally drained that it prevents me from contributing to my family, and too stressed to do the things I enjoy), and behavioral-based (i.e., the problem-solving behaviors I use in my job are not effective in resolving problems at home, behaviors that are effective and necessary for me at work would be counterproductive at home, the behaviors I perform that make me effective at work do not help me to be a better parent and spouse) work interference with family. Cronbach’s alpha was 0.89.

#### 3.2.3. Life Satisfaction

We measured life satisfaction with the five-item Satisfaction with Life Scale (i.e., my life is close to my ideal, the conditions of my life are excellent, I am satisfied with my life, I have gotten the important things I want, I would change almost nothing if I could live my life over) [29]. We asked participants to indicate their agreements with the items. Cronbach’s alpha was 0.78.

#### 3.2.4. FSSB

We used the 14-item scale of Hammer et al. [51] and assessed this construct. Respondents were asked to indicate to what extent (i.e., from 1—strongly disagree to 7—strongly agree) their direct managers were supportive in terms of emotional support, role modeling, instrumental support and creative management with regard to their work-family issues. A sample item was “my supervisor is willing to listen to my problems in juggling work and non-work life” (emotional support). Cronbach’s alpha was 0.92.

#### 3.2.5. Self-Reported Sleep Sufficiency

We measured sleep sufficiency using one item [63,64]. The question was “how often during the past four weeks did you get enough sleep to feel rested upon waking up?” Items were rated on a scale ranging from 0 (never) to 100 (very often).

#### 3.2.6. Objectively-Measured Sleep Sufficiency

We followed Crain et al.’s approach [11] to use a smart wristband to objectively assess sleep sufficiency. The wristband included a heart rate monitor in addition to an accelerometer. Both sensors could detect different stages of sleep because the long-range correlations during wakefulness and rapid eyes movement (REM) sleep were caused by the enhanced influence of the brain on the autonomous nervous system and heart rate was one of the representative signals of the autonomous nervous system [65]. Prior studies reported that such device represents a reliable and valid objective measure of sleep duration, as long as not used for the diagnosis of sleep disorders [11,66,67,68,69]. We followed Crain et al. [11] to objectively measure the sleep sufficiency by the average percentage of REM sleep, light sleep and deep sleep over the total minutes on bed per night.

#### 3.2.7. Control Variables

We included the following control variables in the analyses: age, gender (1 for female and 0 for male), marriage (1 for married and 0 for otherwise), number of children, and educational level (0 for without college degree, 1 for with college degree and 2 for with post-graduate degree).

### 3.3. Descriptive Statistics

Descriptive statistics and correlations of all study variables were presented in Table 1. We found that FRO and WIF were negatively associated with life satisfaction and both sleep sufficiency. FSSB was positively associated with FRO and WIF but negatively associated with life satisfaction and sleep sufficiency. Finally, people with higher education level tended to report a higher level of sleep sufficiency.

## 4. Analyses and Results

### Hypotheses Testing

Although we employed a multiple-wave research design, it was important to test whether the common method bias [69] might occur in this study. We followed MacKenzie and Podsakoff [70]. We added a latent factor called common latent factor (CLF) which was loaded on all observed variables. Then we constrained all new paths to the same regression weight and set the variance of CLF to one to avoid unidentified model. We found that there was no change in the regression weight of unconstrained paths before and after adding CLF. The result indicated that our model was not affected by common method bias.

In addition, we tested the construct validity of our research model with different settings. We first estimated a one-factor CFA model (Model 1) in which all items loaded on one latent variable. As shown in Table 2, the model yielded poor model fit. We estimated a two-factor CFA (Model 2) in which the predictors of WIF and FRO were loaded on one latent factor and the indicators of life satisfaction, self-reported sleep sufficiency and objectively-measured sleep sufficiency were loaded on another latent factor. Then, we conducted a CFA model (Model 3) that separated life satisfaction and sleep and combined only WIF and FRO, the model fit of which also showed poor results. Finally, we tested Model 4, a five-factor CFA model, which (IFI = 0.93, CFI = 0.93, RMSEA = 0.06) fit the data significantly better than the other models. We conducted the Chi-squared tests with these models. The results shown in Table 3 confirmed that Model 4 (with the construct structure of our research model) outperformed the other models in terms of goodness of fit.

Table 4 shows that the path between FRO and life satisfaction was −0.43 (*p* < 0.01) and the one between WIF and life satisfaction was −0.19 (*p* < 0.05), which supported hypotheses H1 and H2. Furthermore, life satisfaction was significantly positively related to both self-reported sleep sufficiency (0.22, *p* < 0.05) and objectively-measured sleep sufficiency (0.23, *p* < 0.01). Thus, hypotheses H3a and H3b were supported.

Then, we assessed the mediation role of life satisfaction by focusing on the indirect effects of WIF and FRO on two sleep variables [71]. We followed Rucker’s approach [71] to add direct paths from FRO and WIF to the two sleep sufficiency variables respectively (Model 5). We estimated Model 5 with a bootstrapping (5000 times). The direct effect of WIF to objective-measured sleep sufficiency was significantly negative (−0.19, *p* < 0.01), while the other three additional paths were not statistically significant. In addition, we followed Rucker et. al. [71] to calculate the size of the mediation effects by computing the product of the two indirect paths respectively. The results were shown in Table 5. Based on the criteria of Shrout and Bolger [72], the mediation effect size of life satisfaction between the nexus of FRO and sleep sufficiency was medium while that of life satisfaction between the nexus of WIF and sleep sufficiency was small. These consistent results led to a conclusion that life satisfaction cannot mediate the impact of WIF on both subjectively-reported and objectively-measured sleep sufficiency.

Lastly, we applied two methods to examine the moderating effect of FSSB. We first generated two interaction variables by multiplying FSSB (aggregated from its items by taking arithmetic mean) and FRO (aggregated score) and WIF (aggregated score) respectively. We estimated a full SEM (Model 6) by including these two interaction variables in our research model. Results shown in Table 2 indicated that Model 6 fit the data quite well. But the paths of both interaction variables were not statistically significant (see Table 4).

To conduct a robustness check, we followed Blunch’s approach [73], by dividing the full sample into high FSSB group and low FSSB group by the median and then re-estimating our research model with two sub-samples (Model 7). The paths between FRO to life satisfactions and between WIF to life satisfaction were also not statistically different between these two groups. Finally, we conducted the chi-squared tests to compare Model 7 to a full SEM (Model 8) in which we constrained the path from FRO to life satisfaction to be equal in both groups, to a full SEM (Model 9) in which we constrained the path from WIF to life satisfaction to be equal in both groups, and to a full SEM (Model 10) in which we constrained both paths to be equal in both groups respectively. Results shown in Table 3 confirmed that Model 8–10 were not statistically different from Model 7, which confirmed the moderating effect of FSSB was trivial. Therefore, the results of both moderating effect tests were consistent so that we concluded that Hypothesis H4 was not supported.

## 5. Discussion

This study tested the relationships among FRO, WIF, life satisfaction and sleep sufficiency in the construction industry using the framework of COR theory. The findings enhanced our understanding on how the intersections between work and family influence people’s life and sleep.

### 5.1. Theoretical Implications

Our research findings offered important contributions to the extant literature. First, this study offered unique insight into the role in which life satisfaction plays. Our findings confirmed that life satisfaction actually played a medium-sized mediating role in the FRO-sleep nexus and therefore provided an empirical evidence to support the theoretical perspective [31] on FRO and life satisfaction, which may have important implications in the domain of public health.

Second, our study advanced our understanding on the limits of FSSB [74,75], as our findings suggested that FSSB was not helpful in alleviating the threats of FRO and WIF on life satisfaction in the construction industry. De Jonge and Dormann [76] argue that the effect of FSSB are more significant when stressors, resources and strains all match in terms of being cognitive, emotional, or physical. This study confirmed that the benefits of FSSB were not universal but were subject to occupational circumstances. In China, as infrastructure demands (e.g., housing and railway construction) have surged to meet the pace of modernization [77], and most construction professionals are obligated to stay at constructions sites for weeks or even months [78]. Long and inflexible work hours, unsafe working conditions and separation from family become the fundamental nature of their occupation, which can hardly be improved by a particular supervisor. That is to say, although supervisors may adjust work assignments to support employees’ family needs, construction professionals may feel stressed because the resources they lose (time, safety, valued family role) are uncompensated. Construction professionals have to always fight with physical exhaustion and to cope with the stress in both workplace and families. Thus, the moderating mechanisms of FSSB in the Chinese construction industry may not be substantial.

Finally, our study reported that WIF has a negative, direct effect on objectively-measured sleep sufficiency. This finding indicated the workaholic culture in the Chinese construction industry may have serious work-exhaustion-related consequences, as work domain occupies most of the resources of construction professionals. They may have little time spending with families or may bring negative emotions to their family domains. The loss of resources is likely to result in strain which prevents construction professionals from achieving high life satisfaction and from attaining adequate amounts of sleep. In short, our study extended the empirical progression of work-family research to wider industry contexts [79] and served as a useful starting point to establish the generalizability of work-family research in the construction industry.

### 5.2. Practical Implications

Our study had implications for construction firms. Our findings indicated that FSSB is not a useful managerial tool to alleviate the impact of FRO and WIF on life satisfaction. Managers may provide substantial help (e.g., a mandatory paid-time-off policy, vocation vouchers, compensatory time) and break workaholic practices in China. Managers also need to revise their definition of hard work by establishing a work-life-balance friendly performance grading system, a system that evaluates output and productivity over the perceived effort and time construction professionals put in.

### 5.3. Limitations and Future Research Directions

Since FSSB was not a statistically significant moderator in this study, future research should explore other potential moderating variables that could alter the links between FRO and WIF and life satisfaction respectively. We suggest that future researches focus on one specific policy (for example, paid leave) to figure out a practical way to help employees in relieving pressure and promoting life satisfaction as well as sleep.

In addition, we included five basic characteristic variables as control variables in the model. However, it is important for future studies to shed more light on the impact of social-economic factors such as gender inequality and age on life satisfaction and sleep problem, as previous studies highlight that caregivers (usually females) [15,80,81,82,83,84,85] and older people [80,86,87] usually suffer more from sleep disturbances.

Finally, we used a sample of construction professional from the southwest region of China, which might limit the generalizability of our findings in other regions or countries. Future studies may conduct cross-cultural comparisons to examine the potential impact of culture differences on our research model.

## Figures and Tables

**Table 1:** Descriptive statistics and correlation matrix.

	Mean	SD	1	2	3	4	5	6	7	8	9	10
1. FRO	2.18	0.74	0.92									
2. WIF	2.45	0.72	0.14 **	0.89								
3. Life Satisfaction	3.96	0.48	−0.40 ***	−0.26 ***	0.78							
4. FSSB	2.42	0.64	0.26 ***	0.22 ***	−0.48 ***	0.92						
5. Self-reported Sleep Sufficiency	82.84	5.36	−0.08	−0.15 **	0.19 ***	−0.30 ***						
6. Objectively-measured Sleep Sufficiency	346.79	46.44	−0.04	−0.22 ***	0.17 **	−0.20 ***	0.86 ***					
7. Age	32.22	6.80	−0.06	−0.03	0.02	0.06	−0.03	0.02				
8. Gender	0.52	0.50	−0.05	0.14 *	−0.06	−0.01	−0.05	−0.04	−0.20 ***			
9. Marriage	0.82	0.42	−0.09	−0.02	0.00	0.06	−0.09	−0.07	0.44 ***	−0.02		
10. Children	0.76	0.48	−0.08	−0.02	0.03	−0.08	−0.03	−0.02	0.34 ***	−0.04	0.45 ***	
11. Education	1.09	0.46	−0.06	−0.05	0.00	−0.10	0.19 ***	0.15 **	−0.08	0.00	−0.08	0.07

Note: the figures in italic and bold are the Cronbach’s alphas. ***: *p* < 0.01; **: *p* < 0.05.

**Table 2:** Measures of model fit for all estimated models.

Model	CMIN/DF	GFI	CFI	IFI	RMSEA	SRMR
1 (One-Factor)	3.39	0.61	0.61	0.63	0.11	0.12
2 (Two-Factor)	2.93	0.66	0.69	0.7	0.1	0.12
3 (Three-Factor)	2.23	0.6	0.63	0.66	0.08	0.13
4 (Research Model)	1.58	0.85	0.93	0.93	0.06	0.07
5 (Direct Paths Added)	1.57	0.86	0.93	0.93	0.06	0.07
6 (Interactions)	1.77	0.82	0.91	0.91	0.06	0.09
7 (No Constrains)	1.53	0.76	0.87	0.87	0.05	0.09
8 (FRO → LS Constrained)	1.53	0.76	0.87	0.87	0.05	0.09
9 (WIF → LS Constrained)	1.53	0.76	0.87	0.87	0.05	0.08
10 (Both Paths Constrained)	1.53	0.76	0.87	0.87	0.05	0.09

**Table 3:** Chi-squared test of nested SEM models.

Model Comparison	ΔCMIN	Δdf	*p*-Value
M1 vs. M2	319.961	1	<0.01
M2 vs. M3	126.25	20	<0.01
M3 vs. M4	659.385	267	<0.01
M7 vs. M8	0.764	1	>0.1
M7 vs. M9	1.679	1	>0.1
M7 vs. M10	3.125	2	>0.1

**Table 4:** Results of path coefficients.

Path	M4	M5	M6
FRO → LS	−0.43 ***	−0.42 ***	3.68 *
WIF → LS	−0.19 **	−0.18 **	0.68
LS → Self Sleep Suff.	0.23 ***	0.22 **	0.24 ***
LS → Obj. Sleep Suff.	0.22 **	0.21 **	0.22 ***
FRO → Self Sleep Suff.		0.02	
FRO → Obj. Sleep Suff.		0.07	
WIF → Self Sleep Suff.		0.11	
WIF → Obj. Sleep Suff.		−0.19 **	
FSSB*FRO → LS			−5.29
FSSB*WIF → LS			−1.27
AGE → LS	0.01	0.01	0.01
GENDER → LS	−0.02	−0.02	0.05
MARRIAGE → LS	−0.06	−0.05	−0.07
CHILDREN → LS	−0.02	−0.02	−0.03
EDUCATION → LS	0.03	0.03	−0.02

***: *p* < 0.01; **: *p* < 0.05.

**Table 5:** Mediation effect size of life satisfaction.

	Self Sleep Suff.	Obj. Sleep Suff.
FRO	−0.09	−0.08
WIF	−0.04	−0.04

## References

[1] Ohayon M.M., Paskow M., Roach A., Filer C., Hillygus D.S., Chen M.C., Langer G., Hirshkowitz M., Consensus N.S.F.S.S. (2019). The National Sleep Foundation’s Sleep Satisfaction Tool. Sleep Health.

[2] Zekun Y. Insomnia Spreads among Young Chinese. http://www.chinadaily.com.cn/a/201903/21/WS5c92fc35a3104842260b1c80.html.

[3] Diekelmann S., Born J. (2010). The memory function of sleep. Nat. Rev. Neurosci..

[4] Benington J.H., Heller H.C. (1995). Restoration of brain energy metabolism as the function of sleep. Prog. Neurobiol..

[5] Irwin M. (2002). Effects of sleep and sleep loss on immunity and cytokines. Brain Behav. Immun..

[6] Franzen P.L., Buysse D.J. (2008). Sleep disturbances and depression: Risk relationships for subsequent depression and therapeutic implications. Dialogues Clin. Neurosci..

[7] Cappuccio F.P., Taggart F.M., Kandala N.-B., Currie A., Peile E., Stranges S., Miller M.A. (2008). Meta-analysis of short sleep duration and obesity in children and adults. Sleep.

[8] Yaggi H.K., Araujo A.B., McKinlay J.B. (2006). Sleep duration as a risk factor for the development of type 2 diabetes. Diabetes Care.

[9] Shahar E., Whitney C.W., REdline S., Lee E.T., Newman A.B., Javier Nieto F., O’CONNOR G.T., Boland L.L., Schwartz J.E., Samet J.M. (2001). Sleep-disordered breathing and cardiovascular disease: Cross-sectional results of the Sleep Heart Health Study. Am. J. Respir. Crit. Care Med..

[10] Rosekind M.R., Gregory K.B., Mallis M.M., Brandt S.L., Seal B., Lerner D. (2010). The cost of poor sleep: Workplace productivity loss and associated costs. J. Occup. Environ. Med..

[11] Crain T.L., Hammer L.B., Bodner T., Kossek E.E., Moen P., Lilienthal R., Buxton O.M. (2014). Work–family conflict, family-supportive supervisor behaviors (FSSB), and sleep outcomes. J. Occup. Health Psychol..

[12] Barnes C.M., Wagner D.T., Ghumman S. (2012). Borrowing from sleep to pay work and family: Expanding time-based conflict to the broader nonwork domain. Pers. Psychol..

[13] Roehling P.V., Moen P., Batt R. (2003). Spillover. Fac. Publ. Hum. Resour. Stud..

[14] Moen P. (2003). It’s about Time: Couples and Careers.

[15] Williams A., Franche R.-L., Ibrahim S., Mustard C.A., Layton F.R. (2006). Examining the relationship between work-family spillover and sleep quality. J. Occup. Health Psychol..

[16] Lallukka T., Rahkonen O., Lahelma E., Arber S. (2010). Sleep complaints in middle-aged women and men: The contribution of working conditions and work–family conflicts. J. Sleep Res..

[17] Alapin I., Fichten C.S., Libman E., Creti L., Bailes S., Wright J. (2000). How is good and poor sleep in older adults and college students related to daytime sleepiness, fatigue, and ability to concentrate?. J. Psychosom. Res..

[18] Spector P.E., Cooper C.L., Poelmans S., Allen T.D., O’driscoll M., Sanchez J.I., Siu O.L., Dewe P., Hart P., Lu L. (2004). A cross-national comparative study of work-family stressors, working hours, and well-being: China and Latin America versus the Anglo world. Pers. Psychol..

[19] Hobfoll S.E. (1989). Conservation of resources: A new attempt at conceptualizing stress. Am. Psychol..

[20] Coverman S. (1989). Role overload, role conflict, and stress: Addressing consequences of multiple role demands. Soc. Forces.

[21] Plant K.M., Sanders M.R. (2007). Predictors of care-giver stress in families of preschool-aged children with developmental disabilities. J. Intellect. Disabil. Res..

[22] Kandel D.B., Davies M., Raveis V.H. (1985). The stressfulness of daily social roles for women: Marital, occupational and household roles. J. Health Soc. Behav..

[23] Bianchera E., Arber S. (2007). Caring and sleep disruption among women in Italy. Sociol. Res. Online.

[24] Major V.S., Klein K.J., Ehrhart M.G. (2002). Work time, work interference with family, and psychological distress. J. Appl. Psychol..

[25] Halbesleben J.R., Harvey J., Bolino M.C. (2009). Too engaged? A conservation of resources view of the relationship between work engagement and work interference with family. J. Appl. Psychol..

[26] Pavot W., Diener E. (2008). The satisfaction with life scale and the emerging construct of life satisfaction. J. Posit. Psychol..

[27] Pavot W., Diener E. (2009). Review of the satisfaction with life scale. Assessing Well-Being.

[28] Ernst Kossek E., Ozeki C. (1998). Work–family conflict, policies, and the job–life satisfaction relationship: A review and directions for organizational behavior–human resources research. J. Appl. Psychol..

[29] Diener E., Emmons R.A., Larsen R.J., Griffin S. (1985). The satisfaction with life scale. J. Personal. Assess..

[30] Gilman R., Huebner S. (2003). A review of life satisfaction research with children and adolescents. Sch. Psychol. Q..

[31] Strine T.W., Chapman D.P., Balluz L.S., Moriarty D.G., Mokdad A.H. (2008). The associations between life satisfaction and health-related quality of life, chronic illness, and health behaviors among US community-dwelling adults. J. Community Health.

[32] Olson R., Crain T.L., Bodner T.E., King R., Hammer L.B., Klein L.C., Erickson L., Moen P., Berkman L.F., Buxton O.M. (2015). A workplace intervention improves sleep: Results from the randomized controlled Work, Family, and Health Study. Sleep Health.

[33] Kossek H., Kossek E. Family Supportive Supervisor Behaviors (FSSB) Training Manual. https://projects.iq.harvard.edu/files/wfhn/files/fssb_training_manual10_13.pdf.

[34] Lee J., Mahendra S., Alvarez P.J. (2010). Nanomaterials in the construction industry: A review of their applications and environmental health and safety considerations. ACS Nano.

[35] Chi S., Han S., Kim D.Y. (2012). Relationship between unsafe working conditions and workers’ behavior and impact of working conditions on injury severity in US construction industry. J. Constr. Eng. Manag..

[36] Leung M.-y., Chan I.Y.S., Yu J. (2012). Preventing construction worker injury incidents through the management of personal stress and organizational stressors. Accid. Anal. Prev..

[37] Lingard H., Brown K., Bradley L., Bailey C., Townsend K. (2007). Improving employees’ work-life balance in the construction industry: Project alliance case study. J. Constr. Eng. Manag..

[38] Liu J.Y., Low S.P. (2011). Work–family conflicts experienced by project managers in the Chinese construction industry. Int. J. Proj. Manag..

[39] Hobfoll S.E. (2001). The influence of culture, community, and the nested-self in the stress process: Advancing conservation of resources theory. Appl. Psychol..

[40] Kahn R.L., Wolfe D.M., Quinn R.P., Snoek J.D., Rosenthal R.A. (1964). Organizational Stress: Studies in Role Conflict and Ambiguity.

[41] Sieber S.D. (1974). Toward a theory of role accumulation. Am. Sociol. Rev..

[42] Goyal M., Singh S., Sibinga E.M., Gould N.F., Rowland-Seymour A., Sharma R., Berger Z., Sleicher D., Maron D.D., Shihab H.M. (2014). Meditation programs for psychological stress and well-being: A systematic review and meta-analysis. JAMA Intern. Med..

[43] Greenhaus J.H., Powell G.N. (2006). When work and family are allies: A theory of work-family enrichment. Acad. Manag. Rev..

[44] Kanfer R., Ackerman P.L., Murtha T.C., Dugdale B., Nelson L. (1994). Goal setting, conditions of practice, and task performance: A resource allocation perspective. J. Appl. Psychol..

[45] Adams G.A., Jex S.M. (1999). Relationships between time management, control, work–family conflict, and strain. J. Occup. Health Psychol..

[46] Tennant C. (2001). Work-related stress and depressive disorders. J. Psychosom. Res..

[47] Åkerstedt T., Kecklund G., Axelsson J. (2007). Impaired sleep after bedtime stress and worries. Biol. Psychol..

[48] Kamel N.S., Gammack J.K. (2006). Insomnia in the elderly: Cause, approach, and treatment. Am. J. Med..

[49] American Academy of Sleep Medicine (2015). Study links lower life satisfaction to sleep problems during midlife. Science News.

[50] Axelrod J., Reisine T.D. (1984). Stress hormones: Their interaction and regulation. Science.

[51] Hammer L.B., Kossek E.E., Yragui N.L., Bodner T.E., Hanson G.C. (2009). Development and validation of a multidimensional measure of family supportive supervisor behaviors (FSSB). J. Manag..

[52] Thomas L.T., Ganster D.C. (1995). Impact of family-supportive work variables on work-family conflict and strain: A control perspective. J. Appl. Psychol..

[53] Allen T.D. (2001). Family-supportive work environments: The role of organizational perceptions. J. Vocat. Behav..

[54] Grandey A.A., Cropanzano R. (1999). The conservation of resources model applied to work–family conflict and strain. J. Vocat. Behav..

[55] Brislin R.W. (1970). Back-translation for cross-cultural research. J. Cross Cult. Psychol..

[56] Min H., Park J., Kim H.J. (2016). Common method bias in hospitality research: A critical review of literature and an empirical study. Int. J. Hosp. Manag..

[57] Meade A.W., Craig S.B. (2012). Identifying careless responses in survey data. Psychol. Methods.

[58] Huang J.L., Curran P.G., Keeney J., Poposki E.M., DeShon R.P. (2012). Detecting and deterring insufficient effort responding to surveys. J. Bus. Psychol..

[59] Breslau N., Roth T., Rosenthal L., Andreski P. (1996). Sleep disturbance and psychiatric disorders: A longitudinal epidemiological study of young adults. Biol. Psychiatry.

[60] Thiagarajan P., Chakrabarty S., Taylor R.D. (2006). A confirmatory factor analysis of Reilly’s Role Overload Scale. Educ. Psychol. Meas..

[61] Matthews R.A., Bulger C.A., Barnes-Farrell J.L. (2010). Work social supports, role stressors, and work–family conflict: The moderating effect of age. J. Vocat. Behav..

[62] Carlson D.S., Kacmar K.M., Williams L.J. (2000). Construction and initial validation of a multidimensional measure of work–family conflict. J. Vocat. Behav..

[63] Buxton O.M., Cain S.W., O’Connor S.P., Porter J.H., Duffy J.F., Wang W., Czeisler C.A., Shea S.A. (2012). Adverse metabolic consequences in humans of prolonged sleep restriction combined with circadian disruption. Sci. Transl. Med..

[64] Buxton O.M., Quintiliani L.M., Yang M.H., Ebbeling C.B., Stoddard A.M., Pereira L.K., Sorensen G. (2009). Association of sleep adequacy with more healthful food choices and positive workplace experiences among motor freight workers. Am. J. Public Health.

[65] Penzel T., Kantelhardt J.W., Lo C.-C., Voigt K., Vogelmeier C. (2003). Dynamics of heart rate and sleep stages in normals and patients with sleep apnea. Neuropsychopharmacology.

[66] Ancoli-Israel S., Cole R., Alessi C., Chambers M., Moorcroft W., Pollak C.P. (2003). The role of actigraphy in the study of sleep and circadian rhythms. Sleep.

[67] Buxton O.M., Klein L.C., Whinnery J., Williams S., McDade T., Grzywacz J.G., Demerouti E. (2013). Biomarkers in work and family research. Current Issues in Work and Organizational Psychology—New Frontiers in Work and Family Research.

[68] Marino E., Lugni C., Borri C. (2013). The role of the nonlinear wave kinematics on the global responses of an OWT in parked and operating conditions. J. Wind Eng. Ind. Aerodyn..

[69] Podsakoff P.M., MacKenzie S.B., Lee J.-Y., Podsakoff N.P. (2003). Common method biases in behavioral research: A critical review of the literature and recommended remedies. J. Appl. Psychol..

[70] Podsakoff P.M., MacKenzie S.B., Podsakoff N.P. (2012). Sources of method bias in social science research and recommendations on how to control it. Annu. Rev. Psychol..

[71] Rucker D.D., Preacher K.J., Tormala Z.L., Petty R.E. (2011). Mediation analysis in social psychology: Current practices and new recommendations. Soc. Personal. Psychol. Compass.

[72] Shrout P.E., Bolger N. (2002). Mediation in experimental and nonexperimental studies: New procedures and recommendations. Psychol. Methods.

[73] Blunch N. (2012). Introduction to Structural Equation Modeling Using IBM SPSS Statistics and AMOS.

[74] Kossek E.E., Pichler S., Bodner T., Hammer L.B. (2011). Workplace social support and work–family conflict: A meta-analysis clarifying the influence of general and work–family-specific supervisor and organizational support. Pers. Psychol..

[75] Jawahar I., Stone T.H., Kisamore J.L. (2007). Role conflict and burnout: The direct and moderating effects of political skill and perceived organizational support on burnout dimensions. Int. J. Stress Manag..

[76] De Jonge J., Dormann C. (2006). Stressors, resources, and strain at work: A longitudinal test of the triple-match principle. J. Appl. Psychol..

[77] Chan A.P., Lam P.T., Chan D.W., Cheung E., Ke Y. (2010). Critical success factors for PPPs in infrastructure developments: Chinese perspective. J. Constr. Eng. Manag..

[78] Xu L.D., Xu E.L., Li L. (2018). Industry 4.0: State of the art and future trends. Int. J. Prod. Res..

[79] Powell G.N., Francesco A.M., Ling Y. (2009). Toward culture-sensitive theories of the work–family interface. J. Organ. Behav..

[80] Gaspar M.O. (2013). The modernisation process through the perceptions of work–family in Spain and Great Britain. Eur. Soc..

[81] Hislop J., Arber S. (2003). Sleepers wake! The gendered nature of sleep disruption among mid-life women. Sociology.

[82] Hislop J., Arber S. (2003). Understanding women’s sleep management: Beyond medicalization-healthicization?. Sociol. Health Illn..

[83] Hislop J., Arber S. (2006). Sleep, gender and aging. Age Matters Realigning Fem. Think.

[84] Meadows R. (2005). The ‘negotiated night’: An embodied conceptual framework for the sociological study of sleep. Sociol. Rev..

[85] Williams S.J. (2002). Sleep and health: Sociological reflections on the dormant society. Health.

[86] Lyonette C., Crompton R., Wall K. (2007). Gender, occupational class and work–life conflict: A comparison of Britain and Portugal. Community Work Fam..

[87] Tobío C. (2001). Working and mothering-women’s strategies in Spain. Eur. Soc..

